# Assessment of combination therapy in BALB/c mice injected with carbapenem-resistant Enterobacteriaceae strains

**DOI:** 10.3389/fmicb.2015.00999

**Published:** 2015-09-22

**Authors:** Noor A. Salloum, Kohar Annie B. Kissoyan, Sukayna Fadlallah, Katia Cheaito, George F. Araj, Rima Wakim, Souha Kanj, Zeina Kanafani, Ghassan Dbaibo, Ghassan M. Matar

**Affiliations:** ^1^Department of Experimental Pathology, Immunology and Microbiology, Center for Infectious Diseases Research, Faculty of Medicine, American University of BeirutBeirut, Lebanon; ^2^Pathology and Laboratory Medicine, Center for Infectious Diseases Research, Faculty of Medicine, American University of BeirutBeirut, Lebanon; ^3^Pediatrics and Adolescent Medicine, Center for Infectious Diseases Research, Faculty of Medicine, American University of BeirutBeirut, Lebanon; ^4^Internal Medicine, Center for Infectious Diseases Research, Faculty of Medicine, American University of BeirutBeirut, Lebanon

**Keywords:** Enterobacteriaceae, carbapenem resistance, carbapenemase, *in vivo*, combination therapy, gene transcript

## Abstract

Monotherapeutic options for carbapenem resistant infections are limited. Studies suggest that combination therapy may be associated with better outcomes than monotherapies. However, this is still controversial. This study assessed, the efficacy of combination therapy against carbapenem resistant Enterobacteriaceae harboring singly various extended spectrum beta lactamase or carbapenemase encoding genes. Thus, four isolates harboring either *bla*_CTXM-15_, *bla*_CTXM-15_ and *bla*_OXA-48_, *bla*_NDM-1_, or *bla*_KPC-2_ genes were selected for testing. Minimal inhibitory concentration was determined by broth dilution method. Gene transcript levels on single and combined treatments were done *in vitro* and *in vivo* by qRT-PCR. Assessment of treatments was done in BALB/c mice according to a specific protocol. As such, the qRT-PCR revealed a significant decrease of transcript levels in all isolates upon using rifampicin or tigecycline, singly or in combination with colistin. However, variable levels were obtained using colistin singly or in combination with meropenem or fosfomycin. *In vivo* assessment showed that all combinations used were effective against isolates harboring *bla*_CTXM-15_, *bla*_OXA-48_, and *bla*_NDM-1_. Conversely, the most significant combination against the isolate harboring *bla*_KPC-2_ gene was colistin with either carbapenem, fosfomycin, or kanamycin. As a conclusion, combination therapy selected based on the type of carbapenemase produced, appeared to be non-toxic and might be effective in BALB/c mice. Therefore, the use of a rationally optimized combination therapy might lead to better results than monotherapy, however, clinical trials are needed for human consumption.

## Introduction

During the past decade, the emergence of multidrug resistant Enterobacteriaceae, namely the third generation cephalosporins increased significantly (Nordmann et al., 2012), leading to the use of carbapenems that represent the primary treatment of choice for such infections (El-Srougy et al., 2012; Nordmann et al., 2012). Nevertheless, the overuse of these antimicrobial agents led to the development of carbapenem resistance (El-Herte et al., 2012; Nordmann et al., 2012; Baroud et al., 2013), such as the resistance by broad-spectrum beta-lactamase production (such as CTX-M or AmpC enzymes) in conjunction with eﬄux pump or outer membrane impermeabilities (such as OmpC/OmpF) and/or the production of carbapenemases (such as KPC-2, OXA-48, or NDM-1 enzymes; Nordmann et al., 2012; Tängdén, 2012; Baroud et al., 2013; Satlin et al., 2014). Most antibacterial agents cross the outer membrane of bacteria through the OmpF and OmpC porin proteins; this holds true for the Enterobacteriaceae family and the β-lactams as well (Pagès et al., 2008; Delcour, 2009; Tsai et al., 2011). Since the β-lactams enter through these porins, it is easily understandable that when the porin permeability decreases, the entry of the β-lactams would decrease as well. Impermeability results when the porin expression is modified, attributable to a decrease in the level of expression of a porin, or a mutation in the porin channel resulting in its non-functionality (Pagès et al., 2008). As a result in the decreased entrance of the β-lactams to the inner cellular space, decreased susceptibility ranges for cephalosporins and carbapenems in Enterobacteriaceae can occur. Carbapenem resistance is often associated with resistance to other classes of antimicrobial agents (Tängdén, 2012), narrowing the treatment options against these multi-drug resistant bacteria. Subsequently, treatment alternatives are limited to colistin, tigecycline, rifampicin, and fosfomycin. (El-Herte et al., 2012; El-Srougy et al., 2012; Tängdén, 2012; Perez and Van Duin, 2013; van Duin et al., 2013). Reports of associated nephro/neuro-toxicity (El-Srougy et al., 2012; Perez and Van Duin, 2013; Satlin et al., 2014), and increased emergence of resistance (Lim et al., 2011; Tängdén, 2012; Satlin et al., 2014) marked their use as controversial and not recommended, especially when used as monotherapy (Lim et al., 2011; Tängdén, 2012). It is currently debatable whether using these antibacterial agents in combination therapy is more advantagous than monotherapy.

Different studies emphasize the importance of using these antimicrobial agents in combination therapy to overcome carbapenem resistance (El-Herte et al., 2012; El-Srougy et al., 2012; Tängdén, 2012; van Duin et al., 2013) especially with limited of prospects in developing new antimicrobial agents (Kmeid et al., 2013), however, the results are still controversial (Tängdén, 2012; Kmeid et al., 2013; Satlin et al., 2014).

This study attempted to assess the effect of combination therapy, both *in vitro* and *in vivo*, in order to recommend potential establishment of effective regimens against carbapenem resistance in Enterobacteriaceae. In addition, we attempted to assess the transcript levels of the carbapenem resistance encoding genes in response to various selected treatment options, *in vitro* and *in vivo*, in order to determine whether certain carbapenemase encoding genes are inducible by the administered antimicrobial agents singly or in combination.

## Materials and Methods

### Clinical Isolates

Two carbapenem resistant *Klebsiella pneumoniae* isolates (IMP33 and IMP216) harboring respectively the *bla*_CTXM-15_ and *bla*_NDM-1_ and one carbapenem resistant *Escherichia coli* isolate (IMP53) harboring the *bla*_OXA-48_ gene along with *bla*_CTXM-15_ gene, obtained from clinical specimens at a tertiary care facility in Beirut, Lebanon, were used in this study (Baroud et al., 2013). A fourth isolate, a *Salmonella* spp. (KPC) harboring the *bla*_KPC-2_ gene, from the Centers for Disease Control and Prevention (CDC) was also included.

### Animal Testing

BALB/c mice were used in this study. All BALB/c mice were obtained from the Animal Care Facility of the University after the approval of the Institutional Animal Care and Use Committee (IACUC). A total number of 416 adult female BALB/c mice, ranging between 6 and 8 weeks-old and weighing between 20 and 30 g, were used. The mice were handled according to “Guide for the Care and Use of Laboratory Animals” (National Research Council [USA], 2011). A total of 80 mice (20 mice/isolate) were used to determine the LD_50_ for each of the isolates IMP33 (*bla*_CTX-M-15_), IMP53 (*bla*_CTX-M-15_ and *bla*_OXA-48_), IMP216 (*bla*_NDM-1_), and KPC (*bla*_KPC-2_) as described by Nowotny (1971). A total of 48 mice were used for the detection of gene transcript levels on single and combined treatments *in vivo* by qRT-PCR. And finally a total of 288 was used for the assessment of combination therapy *in vivo*.

### Bacterial Identification and Susceptibility Testing

Confirmation of the isolates to the species level was performed by API20E kit (Biomérieux, SA13280 Marcy-l’Etoile, France). Antimicrobial susceptibility testing by minimum inhibitory concentration (MIC) was performed by the broth dilution method, and were interpreted according to the Clinical and Laboratory Standards Institute (CLSI) guidelines Clinical and Laboratory Standards Institute [CLSI] (2013), for imipenem, ertapenem, meropenem, fosfomycin, kanamycin, and ceftazidime. Whereas, colistin and tigecycline were interpreted according to the (The European Committee on Antimicrobial Susceptibility Testing [EUCAST], 2014). *E. coli* ATCC 25922 served as a quality control strain.

### Polymerase Chain Reaction

Genomic DNA extraction of the four isolates was performed using the Illustra Bacteria Genomic Prep Mini Spin Kit (GE Healthcare, UK), according to the manufacturer’s instructions and Baroud et al. (2013). The four isolates were tested for the presence of *bla*_TEM-1,_ *bla*_CTX-M-15,_ *bla*_OXA-48,_ *bla*_NDM-1,_ *bla*_KPC-2,_ *omp-*C, and *omp-*F. The primers (Thermo Scientific, Inc., USA) are as follows: TEM-1 Primer: F: 5′-ATGAGTATTCAACATTTCCG-3′, R: 5′-CCAATG CTTAATGAGTGAGG-3′, with an amplicon size of 836 base pair (bp). OMP-C Primer: F: 5′-GTTAAAGTACTGTCCCTCCT G-3′, R: 5′-GAACTGGTAAACCAGACCCAG-3′, with an amplicon size of 1086 bp. OMP-F Primer: F: 5′-CAGGTACTGCA AACGCTGC-3′, R: 5′-GTCAACATAGGTGGACATG-3′, with an amplicon size of 953 bp. OXA-48 Primer: F: 5′-TTGGTG GCATCGATTATCGG-3′, R: 5′-GAGCACTTCTTTTGTGATG GC-3′, with an amplicon size of 744 bp. NDM-1 Primer: F: 5′-GGAAACTGGCGACCAACG-3′, R: 5′-ATGCGGGCCGTAT GAGTGA-3′, with an amplicon size of 678 bp. CTX-M-15 Primer: F: 5′-GGTTAAAAAATCACTGCGTC-3′, R: 5′-TTAC AAACCGTCGGTGACGA-3′, with an amplicon size of 874 bp. KPC-2 Primer: F: 5′-GCAGCGGCAGCAGTTTGTTGATT-3′, R: 5′-GTAGACGGCCAACACAATAGGTGC-3′, with an amplicon size of 184 bp. Amplicons were detected by gel electrophoresis, on 1.5% agarose gel (Seakem LE agarose, Lonza, USA), stained with ethidium bromide (Amresco, USA), visualized under UV illumination (Transilluminator, Haakebuchler Instruments, Inc., USA) using Olympus digital camera and Digi-Doc Program (UVP, CA, UK).

### Quantitative Reverse Transcription PCR

#### On *In Vitro* Samples

For *in vitro* samples, for each isolate different conditions were tested, and in each tube: the bacterial suspension (grown in TSB) was used either alone, or added to either the MIC concentration of colistin, rifampicin, tigecycline, or meropenem respectively, or the MIC concentration of colistin, in combination with the MIC concentration of the former antimicrobial agents. The samples were adjusted to have the same bacterial concentrations in all the tubes, a total of 3.33 × 10^5^ CFU/ml. After 18 h of incubation, RNA extraction was performed.

#### On *In Vivo* Samples

A total number of 48 adult female BALB/c mice was used for this part of the experiment. For the *in vivo* experiments, four different protocols for each of the four isolates was used, having 3 mice/protocol. Each mouse was injected with 3x LD50 dose. Mice were injected with either the bacterial suspension alone or the bacterial suspension in addition to either the MIC dose of colistin alone, or the MIC dose of colistin in combination with the MIC dose of either meropenem or fosfomycin. All bacterial injections were given at time zero, while the treatment was given 1 h post-infection. All injections were given intraperitoneally. After 4 h from the antimicrobial agent injection(s), all the mice were euthanized, dissected, and blood was collected by cardiac puncture. The tubes were centrifuged at 800 g for 20 min at 4°C; serum was collected and the RNA was extracted.

RNA extraction for both the *in vitro* and *in vivo* samples were done using the Illustra RNA spin Mini RNA Isolation Kit (GE healthcare, UK) according to the manufacturer’s instructions. Synthesis of cDNA by the QuantiTect^®^ Reverse Transcription Kit (QIAGEN, Germany) from the extracted RNA was used in the quantitative Reverse Transcription PCR (q-RT PCR) using BioRad CFX96 Real Time System, C1000 Thermal Cycler (Germany). The QuantiFast^TM^ SYBER^®^ green PCR kit (Qiagen, Germany) was used (according to the manufacturer’s instructions) to examine the relative expression of the genes of interest, *in vitro* and *in vivo*, in response to the different treatment options. The primers (Thermo Scientific, Ulm, Germany) for the genes *bla*_CTXM-15_ (Xu et al., 2005), *bla*_OXA-48_ (Swayne et al., 2011), *bla*_NDM-1_ (Zheng et al., 2013), and *bla*_KPC-2_ (Swayne et al., 2011) are as previously described. The primers (Thermo Scientific, Inc., USA) used are: CTX-M-15: Forward Primer: 5′-GCGTGATACCACTTCACCTC-3′, Reverse Primer: 5′- TGAAGTAAGTGACCAGAATC-3′, with an amplicon size of 260 bp. OXA-48: Forward Primer: 5′-TTCGGCCACGGAGCAAATCAG-3′, Reverse Primer: 5′-GATGTGGGCATATCCATATTCATCGCA-3′, with an amplicon size of 240 bp. NDM-1 Primer: Forward Primer: 5′-TTGGCG ATCTGGTTTTCC-3′, Reverse Primer: 5′- GGTTGATCTCCTG CTTGA-3′, with an amplicon size of 195 bp. KPC-2: Forward Primer: 5′-GCAGCGGCAGCAGTTTGTTGATT-3′, Reverse Primer: 5′-GTAGACGGCCAACACAATAGGTGC-3′, with an amplicon size of 184 bp. rpoB (reference gene): Forward Primer: 5′-TCGAAACGCCTGAAGGTC-3′, Reverse Primer: 5′- TTGGAGTTCGCCTGAGC-3′, with an amplicon size of 184 bp. The Bio Rad CFX manager software was used to calculate the ratio of transcription level of the respective treatment used to the transcription level of the bacterial suspension alone for each gene in question for its respective isolate, employing the *rpo*B gene as a standard. As a note, the value for each condition is not the quantity of expression, rather it is the relative expression compared to the control, with the control being 1. Therefore, any value less than 1 is considered to be a reduced expression, while any value above 1 is considered as increased expression. The relative value, is automatically calculated by the software where the value of each condition (which is the use of the different antimicrobial agents) is divided by the value of the control (which is in this case the positive control), that is why this value does not have a unit.

Each sample was run in triplicates for both the housekeeping gene (*rpo*B), which is the control gene, and the gene in inquiry (*bla*_CTXM-15_, *bla*_OXA-48_ and *bla*_CTXM-15_, *bla*_NDM-1_, and *bla*_KPC-2_) in their respective isolates. The qRT-PCR conditions were: 1 cycle of 95°C for 15 min, 45 cycles of: 95°C for 10 s, Ta (Annealing Temperature) for 30 s, and 72°C for 20 s.

### Mice Observations

A total of 80 mice (20 mice/isolate) were used to determine the LD_50_ for each of the isolates IMP33 (*bla*), IMP53 (*bla* and *bla*), IMP216 (*bla*), and KPC (*bla*).

To determine the efficacy of combination therapy *in vivo* the bacterial concentration used for the injections was 3x LD50 for the respective isolate. The *in vivo-*MIC equivalent doses were (Rahal et al., 2011), according to this formula:

(1) $$Antimicrobial\,\;\;\;agent\,\;\;\;in\,\;\;vivo\,\;\;MIC\,\;\;\;dose\,\;\;\left(\mu g\right)=\frac{\begin{array}{c} {}[Antimicrobial\,\;agent\,\;\;in\,\;vitro\,\;MIC\,\;\;(\mu g/\mu l)\;\;\times \\ in\,\;\;vitro\,\;\;MIC\,\;\;\;broth\,\;\;volume\,\;\;(\mu l)\;\times \;concentration\\ (CFU)\;\;of\,\;\;the\,\;isolate\,\;\;ad\,\min istered\,\;\;in\,\;\;vivo] \end{array}}{\left[concentration\,\;\;(CFU)\;\;of\,\;\;the\,\;isolate\,\;\;per\,\;\;in\,\;\;vitro\,\;\;MIC\,\;\;reaction\right]};$$

as these were the concentrations of the antimicrobial agents to be injected in the mice. The antimicrobial agents used for the *in vivo* assessment were: colistin sulfate salt, ceftazidime hydrate, meropenem trihydrate, kanamycin sulfate, tigecycline hydrate (Sigma-Aldrich, Co., St. Louis, MO, USA), ertapenem sodium, imipenem monohydrate (Merck & CO., Inc., West Point, PA, USA), and fosfomycin disodium (abcam Biochemicals). There by, 288 mice were divided into four major groups (Groups I–IV) according to the injected bacteria (IMP33, IMP53, IMP216, or KPC respectively). Furthermore, each major group was divided into 12 subgroups (6 mice/subgroup), in order to assess the efficacy of each treatment with the respective bacteria. All injections were given intraperitoneally. Two sub-groups in each major group served as control. Sub-group 1 received only a TSB injection (at time zero) and served as a negative control. While sub-group 2 received the bacterial injection alone and served as a positive control. Bacterial injections were given for subgroups 2–12 (at time zero). Subsequently *in vivo-*MIC doses of single (colistin or rifampicin) or combined (colistin with either, ertapenem, imipenem, meropenem, ceftazidime, tigecycline, rifampicin, kanamycin, or fosfomycin) treatments were given after 1 h post-infection. The monitoring period was 10 days, and the mice were observed for weight loss and survival rate. Blood collected from dead mice was cultured, and API was performed to confirm the cause of the death being the respective bacterial injections.

### Statistical Analysis

Statistical analysis was performed for qRT-PCR by unpaired *t*-test using the Graph-Pad *t*-test calculator. Additionally, the Kaplan–Meier plot was used to estimate the survival rates. *p*-values < 0.05 were considered to be statistically significant.

## Results

### *In Vitro* Antimicrobial Susceptibility and PCR Profiles of the Four Isolates

The results of antimicrobial susceptibility profiles as determined by MIC, while gene distribution by PCR for each of the four isolates are presented in **Tables 1** and **2** respectively.

**Table 1:** Antimicrobial susceptibility profiles of the four isolates as determined by MIC broth dilution.

Antimicrobial agents / Isolate	IMP33 *Klebsiella pneumoniae* (*bla*_CTXM-15_) MIC(μg/ml)	IMP53 *E. coli* (*bla*_CTX-M-15_ and *bla*_OXA-48_) MIC(μg/ml)	IMP216 *K. pneumoniae* (*bla*_NDM-1_) MIC(μg/ml)	KPC *Salmonella* (*bla*_KPC-2_) MIC(μg/ml)
Ceftazidime	128	1024	4096	128
Ertapenem	2	32	512	4
Meropenem	0.125	4	64	4
Imipenem	2	8	1024	4
Fosfomycin	4096	512	>1024	256
Kanamycin	64	128	>16384	32
Tigecycline	8	0.5	4	0.5
Colistin	2	2	128	2
Rifampicin	32	8	128	8


MIC, minimum inhibitory concentration.

**Table 2:** Gene distribution in the four isolates as determined by PCR.

Gene / Isolate	IMP33 *K. pneumonia* (*bla*)	IMP53 *E. coli* (*bla* and *bla*_OXA-48_)	IMP216 *K. pneumonia* (*bla*)	KPC *Salmonella* (*bla*)
*bla*	+	+	+	+
*bla*_CTX-M-15_	+	+	-	-
*bla*_OXA-48_	-	+	-	-
*bla*_NDM-1_	-	-	+	-
*bla*_KPC-2_	-	-	-	+
*omp-*C	+	+	-	+
*omp-*F	-	+	+	-


“+”, positive result; “-”, negative result.

### Quantitative Reverse Transcription PCR

#### On *In Vitro* Samples

**FIGURE 1:**
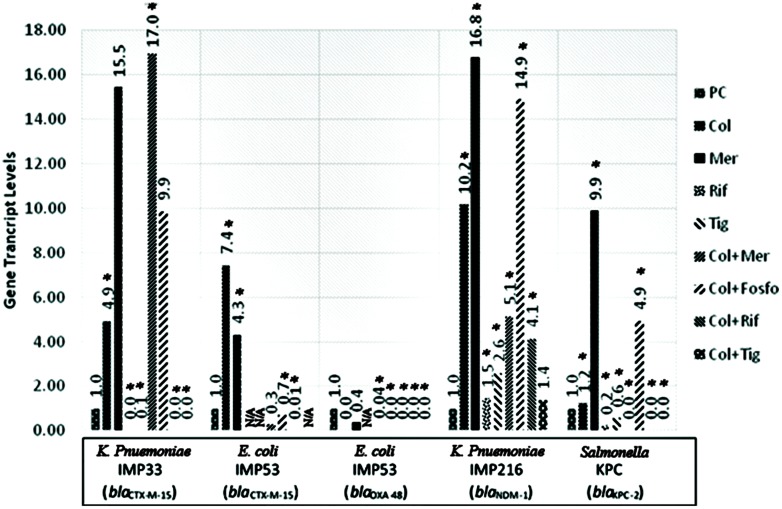
**Gene transcript levels *in vitro* for the genes *bla*_CTX-M-15_, *bla*_CTX-M-15_ and *bla*_OXA-48_, *bla*_NDM-1_, *bla*_KPC-2_ in their respective isolates IMP33, IMP53, IMP216, and KPC treated with Colistin, Meropenem, Rifampicin, Tigecycline, Colistin+Meropenem, Colistin+Fosfomycin, Colistin+Rifampicin, or Colistin+Tigecycline at the MIC levels.** IMP33: colistin: 4.94 (*p* = 0.025), meropenem: 15.54 (*p* = 0.376), rifampicin: 0 (*p* = 0.002), tigecyclin: 0.07 (*p* = 0.038), colistin and meropenem: 16.95 (*p* = 0.012), colistin and fosfomycin: 9.87 (*p* = 0.487), colistin and rifampicin: 0 (*p* = 0.001), colistin and tigecycline: 0 (*p* = 0.025). IMP53 (CTX-M-15): colistin: 7.44 (*p* = 0.042), meropenem: 4.31 (*p* = 0.026), rifampicin: N/A, tigecyclin: N/A, colistin and meropenem: 0.28 (*p* = 0.068), colistin and fosfomycin: 0.69 (*p* = 0.049), colistin and rifampicin: 0.01 (*p* = 0.037), colistin and tigecycline: N/A. IMP53 (OXA-48): colistin: 0 (*p* = 0.875), meropenem: 0.36 (*p* = 0.746), rifampicin: N/A, tigecyclin: 0.04 (*p* = 0.001), colistin and meropenem: 5.14 (*p* = 0.041), colistin and fosfomycin: 14.91 (*p* = 0.002), colistin and rifampicin: 0 (*p* = 0.045), colistin and tigecycline: 0 (*p* = 0.001). IMP216: colistin: 10.20 (*p* = 0.012), meropenem: 16.79 (*p* = 0.046), rifampicin: 1.47 (*p* = 0.049), tigecyclin 2.60 (*p* = 0.002), colistin and meropenem: 5.14 (*p* = 0.050), colistin and fosfomycin: 14.91 (*p* = 0.002), colistin and rifampicin: 4.14 (*p* = 0.002), colistin and tigecycline: 1.38 (*p* = 0.372). KPC: colistin: 1.23 (*p* = 0.005), meropenem: 9.89 (*p* = 0.019), rifampicin 0.23 (*p* = 0.001), tigecyclin 0.56 (*p* = 0.043), colistin and meropenem: 0 (*p* = 0.011), colistin and fosfomycin: 4.93 (*p* = 0.032), colistin and rifampicin: 0 (*p* = 0.012), colistin and tigecycline: 0 (*p* = 0.048). PC, positive control; Col, colistin; Mer, meropenem; Fos, fosfomycin; Rif, rifampicin; and Tig, tigecycline.

The *in vitro* assessment of the effect of combination therapy on gene transcript levels by qRT-PCR, revealed, in all isolates, a decrease in the extended spectrum beta lactamase (ESBL) and carbapenemase encoding gene transcript levels when rifampicin and tigecycline were used individually, or in combination with colistin. Whereby, rifampicin monotherapy or in combination with colistin, *in vitro*, led to the most noticeable decrease in the gene transcript levels of *bla*_CTX-M-15,_ *bla*_OXA-48_, *bla*_NDM-1_, and *bla*_KPC-2_. Also, Tigecycline monotherapy or in combination with colistin, in most of the isolates, resulted in a significant decline in the carbapenemase gene transcript levels. The combination of tigecycline and colistin appears to lower carbapenemase transcript levels than their respective monotherapies. The *in vitro* transcript levels of the ESBL and carbapenemase encoding genes, for the respective isolates harboring the various genes are shown in **Figure 1**.

#### On *In Vivo* Samples

**FIGURE 2:**
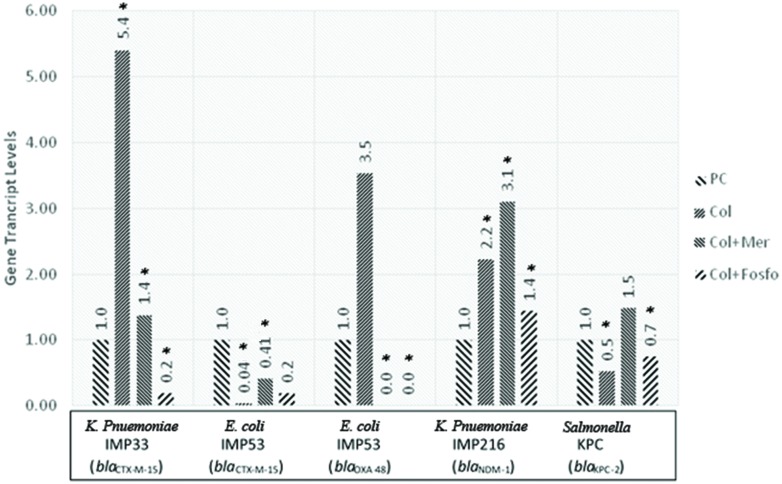
**Gene transcript levels *in vivo* for the genes *bla*_CTX-M-15_, *bla*_CTX-M-15_ and *bla*_OXA-48_, *bla*_NDM-1_, *bla*_KPC-2_ in their respective isolates IMP33, IMP53, IMP216, and KPC treated with Colistin, Colistin+Meropenem, or Colistin+Fosfomycin at the MIC levels.** IMP33: colistin: 5.40 (*p* = 0.053), colistin and meropenem: 1.38 (*p* = 0.038), colistin and fosfomycin: 0.19 (*p* = 0.024). IMP53 (CTX-M-15): colistin: 0.04 (*p* = 0.038), colistin and meropenem: 0.41 (*p* = 0.029), colistin and fosfomycin: 0.19 (*p* = 0.096). IMP53 (OXA-48): colistin: 3.53 (*p* = 0.221), colistin and meropenem: 0 (*p* = 0.041), colistin and fosfomycin: 0 (*p* = 0.049). IMP216: colistin: 2.22 (*p* = 0.043), colistin and meropenem: 3.10 (*p* = 0.021), colistin and fosfomycin: 1.44 (*p* = 0.052). KPC: colistin: 0.53 (*p* = 0.026), colistin and meropenem: 1.49 (*p* = 0.980), colistin and fosfomycin: 0.75 (*p* = 0.042). PC, positive control; Col, colistin; Mer, meropenem; Fos, fosfomycin.

The *in vivo* assessment of the effect of combination therapy on gene transcript levels by qRT-PCR revealed, in most of the isolates, different responses when compared to their respective *in vitro* responses. Moreover, when colistin was used singly or in combination with meropenem or fosfomycin *in vivo*, the qRT-PCR showed variable gene transcript levels for the respective ESBL and carbapenemase encoding genes within the *in vivo* samples. The transcript levels of the ESBL and carbapenemase encoding genes, for the respective isolates harboring the various genes are shown in **Figure 2**.

### Mice Observations

#### *In Vivo*: LD_50_

The LD_50_ of the four isolates IMP33(*bla*_CTXM-15_), IMP53 (*bla*_CTXM-15_ and *bla*_OXA-48_), IMP216 (*bla*_NDM-1_), and KPC (*bla*_KPC-2_) was determined to be 5.62 × 10^5^, 1 × 10^7^, 1.47 × 10^7^, and 1 × 10^8^ CFU respectively.

#### Assessment of the Efficacy of Treatments *In Vivo*

After the 10-days monitoring period, all the mice in negative control sub-groups (no bacteria injected) survived the monitoring period. On the other hand, the survival rate in the positive control sub-groups (only bacteria injected) was 33% for Group I injected with isolate IMP33 (*bla*_CTX-M-15_), 0% in Group II injected with isolate IMP53 (*bla*_CTXM-15_ and *bla*_OXA-48_), 83% for Group III injected with isolate IMP216 (*bla*_NDM-1_), whereas 0% in Group IV injected with isolate KPC (*bla*_KPC-2_), respectively.

**FIGURE 3:**
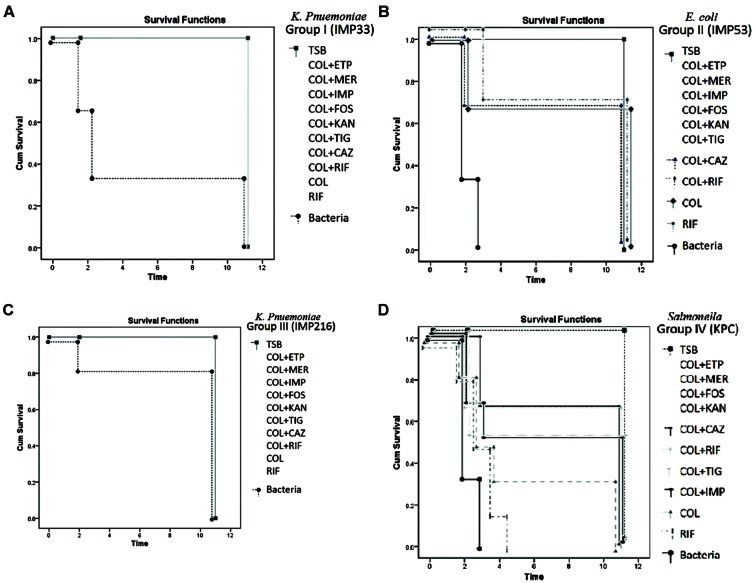
**(A)** Percentage of survivals of Group I injected with isolate IMP33, harboring *bla*_CTX-M-15_ gene, during the monitoring period (*p*-value = 0.049). The concentration of the bacteria injected (3xLD50) = 1.68 × 10^6^ CFU/μl. Concentration of the antimicrobial agents added: COL: 6.744 μg/μl, ETP: 6.744 μg/μl, IMP: 6.744 μg/μl, MER: 0.4215 μg/μl, RIF: 107.904 μg/μl, FOS: 13811.712 μg/μl, KAN: 215.808 μg/μl, TIG: 26.976 μg/μl. **(B)** Percentage of survivals of Group II injected with isolate IMP53, harboring both *bla*_CTX-M-15_ and *bla*_OXA-48_ genes, during the monitoring period (*p*-value = 0.031). The concentration of the bacteria injected (3x LD50) = 3 × 10^7^ CFU/μl. Concentration of the antimicrobial agents added: COL: 120, ETP: 1920 μg/μl, IMP: 480 μg/μl, MER: 240 μg/μl, RIF: 480 μg/μl, FOS: 30720 μg/μl, KAN: 7680 μg/μl, TIG: 30 μg/μl. **(C)** Percentage of survivals of Group III injected with isolate IMP216, harboring *bla*_NDM-1_ gene, during the monitoring period (*p*-value = 0.029). The concentration of the bacteria injected (3x LD50) = 6.48 × 10^6^ CFU/μl. Concentration of the antimicrobial agents added: COL: 11289.6 μg/μl, ETP: 45158.4 μg/μl, IMP: 90316.8 μg/μl, MER: 5644.8 μg/μl, RIF: 11289.6 μg/μl, FOS: 90316.8 μg/μl, KAN: 1445069 μg/μl, TIG: 352.8 μg/μl. **(D)** Percentage of survivals of Group IV injected with isolate KPC, harboring the *bla*_KPC-2_ gene, during the monitoring period (*p*-value = 0.043). The concentration of the bacteria injected (3x LD50) = 3 × 10^8^ CFU/μl. Concentration of the antimicrobial agents added: COL: 1200 μg/μl, ETP: 2400 μg/μl, IMP: 2400 μg/μl, MER: 2400 μg/μl, RIF: 4800 μg/μl, FOS: 4800 μg/μl, KAN: 19200 μg/μl, TIG: 300 μg/μl. TSB, Tryptic Soy Broth; COL, colistin; CAZ, ceftazidime; ETP, ertapenem; IMP, imipenem; MER, meropenem; KAN, kanamycin; FOS, fosfomycin; and TIG, tigecycline.

As for the sub-groups (3–12) in both Groups I and III, injected with isolates IMP33 (*bla*_CTX-M-15_) and IMP216 (*bla*_NDM-1_) respectively, which received an injection of antimicrobial agents singly or in combination, a 100% survival rate was observed. On the other hand, the sub-groups (3–12) in Group II, injected with isolate IMP53 (*bla*_CTXM-15_ and *bla*_OXA-48_) was 80%. While the lowest survival rate of 66% was observed in sub-groups (3–12), in Group IV, injected with isolate KPC (*bla*_KPC-2_). The survival rates according to the treatments for each of the four isolates are shown in **Figures 3A–D**.

During the monitoring period, the average weight of the mice (figures not shown) in the negative control sub-groups for the four isolates increased, while that of the positive control sub-groups (expect for that of Group III) decreased within the first 4 days. As for the other sub-groups in Groups I, II and IV, the mice that survived showed weight regain 5-days post-receiving the respective bacterial and treatment injections. With regard to all the subgroups in Group III that received bacterial injections (with or without treatment) no significant weight loss was observed; rather, the mice showed stability and weight gain.

The API testing confirmed that the cause of death was due to the respective bacterial injections; i.e., *K. pneumonia* for isolates IMP33 (*bla*_CTX-M-15_) and IMP216 (*bla*_NDM-1_), *E. coli* for isolate IMP53 (*bla*_CTXM-15_ and *bla*_OXA-48_), and *Salmonella* spp. for KPC (*bla*_KPC-2_) isolate.

## Discussion

This study attempted to assess the transcript levels of the carbapenem resistance encoding genes in response to various selected treatment options, *in vitro* and *in vivo*, in order to determine whether certain carbapenemase encoding genes are inducible by the administered antimicrobial agents singly or in combination. Based on the qRT-PCR results, rifampicin monotherapy or in combination with colistin, *in vitro*, led to the most noticeable decrease in the gene transcript levels of *bla*_CTX-M-15,_ *bla*_OXA-48_, *bla*_NDM-1_, and *bla*_KPC-2_. This result was expected since rifampicin inhibits RNA synthesis by inhibiting gene transcription (Rahal et al., 2011). Although rifampicin monotherapy resulted in the most efficient transcript inhibition, its use as monotherapy is not recommended due to the high rate of emerging resistance (Kohanski et al., 2010). Consequently, the combination of colistin and rifampicin seems to be a better treatment option.

Similarly, the *in vitro* tigecycline monotherapy or in combination with colistin, in most of the isolates, resulted in a significant decline in the carbapenemase gene transcript levels. This can be explained by the fact that tigecycline inhibits the synthesis of proteins used in catalyzing RNA synthesis (Herbert et al., 2001). The combination of tigecycline and colistin appears to be more effective, leading to lower carbapenemase transcript levels than their respective monotherapies.

*In vitro* and *in vivo* testing of colistin in combination with either meropenem or fosfomycin, or their monotherapies, led to different gene transcript levels in isolates IMP33 (*bla*_CTX-M-15_), IMP216 (*bla*_NDM-1_) and KPC(*bla*_KPC-2_), when compared to their positive controls. This might be explained by the fact that these antimicrobial agents do not inhibit gene transcription like rifampicin, rather act on the bacterial cell wall, and/or by the induction of the survival mode in bacteria to increase the production of the hydrolyzing enzymes (El-Herte et al., 2012). In addition, the *bla*_CTX-M-15_ gene transcript levels in isolates IMP33 and IMP53 were different; this difference might be attributed to the fact that the isolate IMP33 is a *K. pneumoniae*, while the isolate IMP53 is an *E. coli* isolate.

The difference between *in vitro* and *in vivo* results may limit the understanding of the efficacy of combination therapy. These discrepancies could be explained by the fact that *in vivo* conditions can trigger different mechanisms in bacteria (Davies et al., 1988). In addition, the effect of the immune system in the host, the pharmacokinetic and pharmacodynamic parameters of the antimicrobial agents may explain some discrepancies between *in vitro* and *in vivo* (Chen et al., 2011; Polak, 2013). In addition, we have the possibility of experimental variation between the *in vitro* and the *in vivo* experimental setups.

For isolate IMP53 (*bla*_CTX-M-15_ and *bla*_OXA-48_), an evident decrease in *bla*_OXA-48_ gene transcript levels was observed after all treatment modes, both *in vitro* and *in vivo* (except with colistin monotherapy). In fact, the MIC levels of IMP53 (*bla*_CTXM-15_ and *bla*_OXA-48_), are reported as low level resistance to both imipenem and meropenem, and susceptible to colistin. Indeed, even though OXA enzymes confer carbapenem resistance, they exhibit poor carbapenem and cephalosporin hydrolysis activities (Queenan and Bush, 2007). As a result, these antimicrobial treatments might be effective in killing the bacteria, thus explaining the low gene transcript levels. Another reason might be explained by the fact that the plasmid harboring the *bla*_OXA-48_ gene is associated with insertion sequences, which provide the carbapenemase encoding gene with a promoter region that controls its transcription level (Depardieu et al., 2007; El-Herte et al., 2012). The promoter involved in this isolate might not be efficient, reflecting a decrease in the gene transcript levels (Depardieu et al., 2007; El-Herte et al., 2012).

Concerning the efficacy of combination therapy *in vivo*, carbapenem resistance is not necessarily associated with high pathogenicity or virulence (CDC, 2014), which might explain the 100% survival rate observed in Group I injected with isolate IMP33 (*bla*_CTX-M-15_) followed by treatment. Moreover, carbapenem resistance in this isolate is due to ESBL production and porin loss which represents a lesser clinical threat than carbapenemase production (Opazo et al., 2012). As a result, the treatment regimens for such infections caused by ESBL producing isolates having porin loss, in BALB/c mice, extend to include colistin in combination with an adjuvant antimicrobial agent such as a carbapenem, rifampicin, or kanamycin (fosfomycin or tigecycline remain for severe infections).

The advisable treatments for infections caused by OXA-48 producing isolates include: colistin in combination with an adjuvant such as a carbapenem, rifampicin, fosfomycin, or tigecycline. Although there are no definitive clinical data that demonstrate improved outcomes with combination versus monotherapy and one trial suggested that colistin and rifampin combination and colistin monotherapy were equivalent, infections with multidrug resistant Acinetobacter and Enterobacteriaceae are associated with high mortality rates and we are concerned that the use of a single agent is not adequate, particularly since resistance can develop during therapy leaving no therapeutic alternatives (Falagas et al., 2014).

The general concept of colistin used in combination with other agents (versus colistin monotherapy) appears beneficial in the mice infected with virulent CRE strains. However, for drugs that are active against protein synthesis pathways (rifampicin, tigecycline), while appearing to reduce resistance gene expression *in vitro*, were not shown to add clear clinical benefit when used in combination *in vivo*, except perhaps colistin with rifampicin for KPC *Salmonella*.

The total survival rate for Group III injected with isolate IMP216 (*bla*_NDM-1_) was 100%. Isolate IMP216 was identified as extensively drug resistant as it was resistant to all the tested antimicrobial agents; nevertheless, 83% of the mice in the positive control subgroups (3–12) survived. Moreover, there was no significant weight loss observed in these subgroups. In fact, several studies have reported colonization of organisms harboring the *bla*_NDM-1_ gene as part of the fecal flora (Chen et al., 2011; Samuelsen et al., 2011). This might be explained by the fact that antimicrobial resistance can be associated with both a decreased fitness, expressed by impairment of the bacterial growth in the infected host (Andersson, 2006; Samuelsen et al., 2011), and a decreased virulence, represented by diminished invasiveness and higher clearance rates (Skurnik et al., 2013). Furthermore, the most effective treatment option for such infections in BALB/c mice, caused by organisms harboring the *bla*_NDM-1_ gene include, and might be limited to, combination therapy of colistin with either rifampicin, fosfomycin, or tigecycline. It is worth mentioning that it is possible that the two *Klebsiella* isolates (IMP33 and IMP216) used in this study, when injected intraperitoneally, are not as lethal as *E. coli* (IMP53) or *Salmonella* (KPC) isolates given by that route.

Lastly, the mice group that received the KPC isolate (*bla*_KPC-2_) injections followed by treatment, revealed the lowest survival rates of 66%, and the surviving mice presented clinical symptoms of illness with clear weight loss and diarrhea, demonstrating high virulence. This could be due to the fact that this isolate is of a *Salmonella* species. Therefore, based on the survival rates in BALB/c mice, the treatment options for such infections include colistin in combination with a carbapenem, fosfomycin, or kanamycin.

However, the treatment regimens that are not recommended for infections caused by multi-drug resistant Enterobacteriaceae, include the monotherapies of either rifampicin, tigecycline, aztreonam, carbapenem, or colistin, and the combination therapy of a carbapenem with a third generation cephalosporin (Lim et al., 2011; El-Srougy et al., 2012; Tängdén, 2012; Perez and Van Duin, 2013; van Duin et al., 2013).

Based on the overall survival rates in our experiments, it can be concluded that combination therapy is not associated with increased death rates, as reported previously (El-Srougy et al., 2012; Tängdén, 2012; Satlin et al., 2014), especially since treatment with colistin, the selected cornerstone antimicrobial agent in these treatments, has high reports of neuro/nephro-toxicities (Lim et al., 2011; Tängdén, 2012; Kmeid et al., 2013). It should be noted that different species used in this study may exhibit different pathogenic effects, which might explain the different results and values.

## Conclusion

Based on the gene transcript levels and mice survival rates, one generalized regimen cannot be administered as an effective treatment for the various carbapenem resistant isolates; thereby, stressing the importance of phenotypic/genotypic testing and the determination of resistance encoding genes for each isolate. The use of a rationally optimized combination therapy might lead to better results than monotherapy especially in virulent strains. This is due to the fact that monotherapy *in vivo* using the same resistant strains and ertapenem, as we demonstrated in a previous unpublished preliminary study, resulted in poor survival outcome. Moreover, it would noteworthy to indicate that clinical trials are needed to further assess the efficacy of combination therapy in humans.

## Conflict of Interest Statement

The authors declare that the research was conducted in the absence of any commercial or financial relationships that could be construed as a potential conflict of interest.
